# Design, Synthesis and Trypanocidal Evaluation of Novel 1,2,4-Triazoles-3-thiones Derived from Natural Piperine

**DOI:** 10.3390/molecules18066366

**Published:** 2013-05-29

**Authors:** Tatiany Nunes Franklim, Leonardo Freire-de-Lima, Julliana de Nazareth Sá Diniz, José Osvaldo Previato, Rosane Nora Castro, Lucia Mendonça-Previato, Marco Edilson Freire de Lima

**Affiliations:** 1 Departamento de Química, Instituto de Ciências Exatas, Universidade Federal Rural do Rio de Janeiro, BR 465, Km 07, CEP: 23.890-000, Seropédica, RJ, Brazil; E-Mails: tnfranklim@gmail.com (T.N.F.); nora@ufrrj.br (R.N.C.); 2 Cidade Universitária, Ilha do Fundão, Instituto de Biofísica Carlos Chagas Filho, Universidade Federal do Rio de Janeiro, CEP: 21.941-902, Rio de Janeiro, RJ, Brazil; E-Mails: leolima@biof.ufrj.br (L.F.-L.); jullianawinners@gmail.com (J.N.S.D.); previato@biof.ufrj.br (J.O.P.); luciamp@biof.ufrj.br (L.M.-P.)

**Keywords:** *Trypanosoma cruzi*, *Piper nigrum*, antiparasitic drugs, bioisosterism, molecular hybridization

## Abstract

The work reported herein describes the synthesis and the assessment of the trypanocidal activity of thirteen new 1,2,4-triazole-3-thiones obtained from natural piperine, the main constituent of the dry fruits of *Piper nigrum*. It is part of a research program aiming to use abundant and easily available natural products as starting materials for the design and synthesis of new molecules potentially useful as antiparasitic drugs. The variously substituted triazole derivatives were synthesized from the natural amide in four steps with the use of microwave irradiation on overall yields ranging from 32% to 51%. The cyclohexyl substituted derivative showed the best trypanocidal profile on proliferative forms of *Trypanosoma cruzi* (Y strain), with IC_50_s = 18.3 and 8.87 μM against epimastigotes and amastigotes, respectively.

## 1. Introduction

Among the most important natural molecules that have had strong impact on human health during the last century we can mention quinine, extracted from *Cinchona officinales* (Rubiaceae) and penicillin, obtained from *Pennicillium chrysogenum* (Trichocomaceae) through fermentation processes [1]. Another naturally occurring molecule of great importance is taxol, first isolated from *Taxus brevifolia* (Taxaceae). Taxol is an antitumor drug which has application in the treatment of various cancers such as ovarian, lung, melanoma and breast cancer [1]. Thus it may be seen that the natural products occupy a prominent position in drug development, mainly in the chemotherapy of infectious diseases and cancer [1,2,3,4].

**Figure 1:**
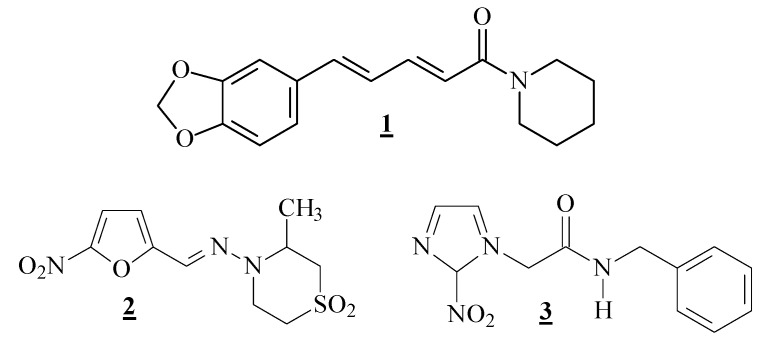
Chemical structures of piperine (**1**), nifurtimox (**2**) and benznidazole (**3**).

When planning to use natural products as drugs or even as raw materials for drug synthesis, there is a limitation due to the fact that special metabolites of plant origin are biosynthesized in small quantities and, normally, isolated in a very laborious way [5]. Another significant limitation to the use of natural products in drug development is the fact that most of them are not obtained from sustainable sources [5]. Natural piperine (**1**, Figure 1) is an exception, due to its abundance and ease of isolation. Although found in different species of *Piper*, piperine is more abundant in the fruits of *Piper nigrum*, from where it can be extracted in yields of about 3%–7% [6,7]. *P. nigrum* sees widespread use in folk medicine in India and in Brazil, its main use being as a seasoning. Due to its great economic importance in different countries, including Brazil, nowadays large areas are cultivated with *Piper nigrum* [7,8]. The chemical composition of black pepper has motivated scientific studies for almost two hundred years since the first report describing the isolation of piperine from *P. nigrum* was published in 1819 by Hans Christian Oersted [8]. Various biological activities have been attributed to piperine, including insecticidal properties and inhibition of liver metabolism, stimulation of melanocyte proliferation**,** and antitumoral activity [7,8,9,10]. Piperine has been described as having toxic activity against different species of protozoa from the Trypanosomatidae family such as *Leishmania donovani* [11], *Leishmania amazonensis* [12], and *Trypanosoma cruzi* [13,14].

Despite the fact that neglected diseases, including tuberculosis, are responsible for 11.4% of the cause of all disabling clinical conditions in the World, only 21 (1.3%) of the 1,556 new drugs registered between 1,975 and 2,004 were developed specifically for the treatment of these infections [15]. These illnesses are known as neglected diseases due to the lack of investment from the pharmaceutical industry in the development of new drugs to treat the patients and also to the shortage of public policies aimed at their prevention [16]. The literature reports some studies concerning the development of new molecules derived from natural products for the treatment of neglected diseases [17]. Our research group has been working during the last years on the discovery of new chemical entities that may be applicable to the treatment of leishmaniasis and Chagas’ disease, using piperine as raw material [12,13,14,18].

Chagas’ disease is an important parasitic infection, endemic in Latin America, which has the hemoflagelated protozoa *Trypanosoma cruzi* as its etiological agent [19,20]. This infection is normally transmitted by feces and urine of triatomine insects while blood feeding on a vertebrate host. The parasite has a complex life cycle with evolutive forms present in vertebrate and invertebrate hosts. The trypomastigote form when inside the insect differentiates into the epimastigote form and, after replication, reaches the posterior insect’s intestinal tract, where it differentiates into the infective metacyclic trypomastigote form. When inside of vertebrate cells, the metacyclic trypomastigote form undergoes differentiation into amastigotes, which after several replicative cycles, cause the cell breakage, being released into the blood stream of the vertebrate where it differentiates into trypomastigotes, which are responsible for the dissemination of the infection [19]. According to the World Health Organization (WHO), some 10 million people are infected with the *T. cruzi*, mainly in Latin America, resulting in an estimated annual death toll of 10,000 [19,20]. Only two drugs are available for the treatment of Chagas disease: benznidazole (**2**) and nifurtimox (**3**) (Figure 1) [19,20]. These compounds are responsible for undesirable side effects, including anorexia, vomiting, peripheral polyneuropathy, and allergic dermopathy. Another matter of great importance is that the efficacy of these drugs in the chronic phase of infection is still controversial [21]. Thus, considerable efforts have being directed to developing new chemotherapeutical agents for treating chagasic patients, especially using natural products as starting materials or even as a source of inspiration [10,22].

This work describes the synthesis and the evaluation of the trypanocidal activity of thirteen new 1,2,4-triazole-3-thiones **9a**–**9m** derived from natural piperine (**1**). The 1,2,4-triazole-3-thiones are five-membered heterocyclic rings present in the structure of numerous compounds with useful and wide-ranging biological activities, including antidepressant [23], antifungal [24], anti-inflammatory [25], antiviral [26] and antitumor effects [27].

## 2. Results and Discussion

### 2.1. Chemistry

**Figure 2:**
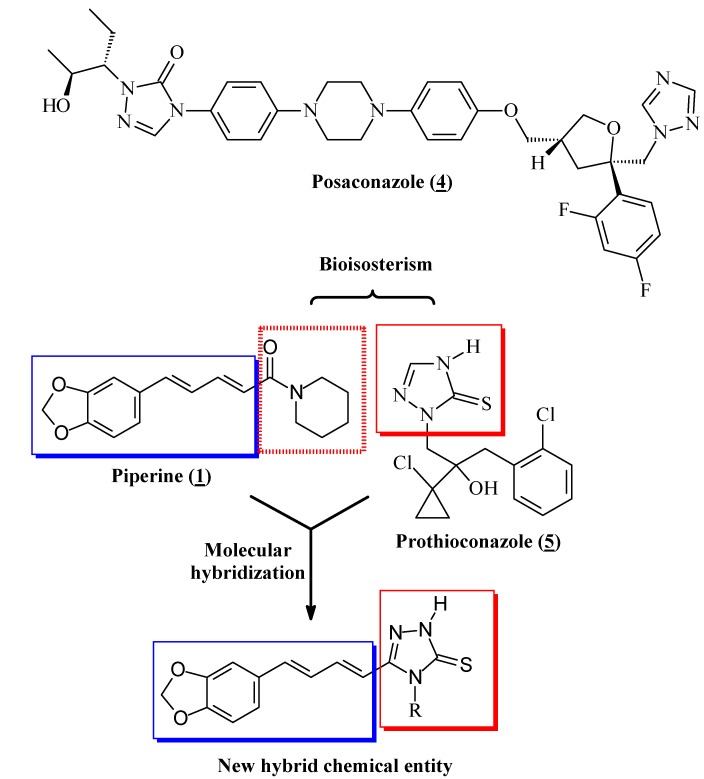
Strategies on the designing of new piperine-triazole hybrid.

When planning new drugs as chemotherapeutic agents for infectious diseases it is necessary to have in mind the possible biochemical targets as well the differences between the parasite`s and host`s cells. Ergosterol is the major sterol component of fungi and also of the *T. cruzi* cell membranes. This lipid plays an important role in the regulation of membrane fluidity and integrity [28]. Azoles are a known class of inhibitors of the cytochrome P-450 dependent sterol 14α-demethylase (CYP51) that promotes the 14α-demethylation of lanosterol into ergosterol. The blockage of this biosynthetic pathway leads to the accumulation of toxic pre-ergosterol lipids in the parasite membrane compromising the cell viability [28]. Posaconazole (**4**, Figure 2) is an example of a broad spectrum anti-fungal azole that has been investigated with respect to its trypanocidal activity. The azole **4** is currently entering phase II clinical trials [29]. Another example of antifungal with the triazole moiety is the prothioconazole (**5**), a 1,2,4-triazole-3-thione and known inhibitor of 14α-demethylase, commercially available for the treatment of plant-pathogenic fungal infections [24]. The nitrogen-containing heterocyclics present in CYP51 inhibitors are important structural features for their coordination to the heme iron and stabilization of the complex enzyme-inhibitor [30]. Having this information in mind the molecular hybridization of piperine and the 1,2,4-triazole nucleus was planned [31], furnishing a series of new compounds where the *N*_4_-substituents the triazole ring were modified changing their electronic and lipophilic profiles in order to allow the investigation of the SAR (Figure 2). The molecular modification approach used on the designing the new derivatives was also based in the bioisosterism strategy [32], since there are different examples in the literature of the bioisosteric replacement of amides by five membered nitrogen-containing rings as triazole, thiadiazole and oxadiazole (Figure 2) [33].

**Scheme 1:**
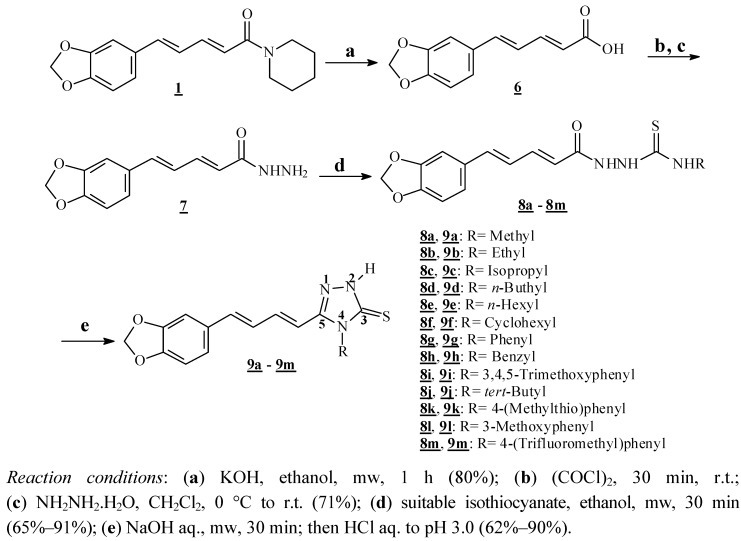
Preparation of new 1,2,4-triazole-2-thiones **9a**–**9m** from piperine (**1**).

Firstly, the isolation of suitable amounts of natural amide was carried out according to a previously described methodology [6]. As shown in Scheme 1, piperic acid (**6**) was obtained in excellent yield by basic hydrolysis of piperine (**1**). Originally this reaction was carried out in 24 h reflux [13], but after improvements on the experimental conditions it could be carried out in 1 h and with 80% yield in a microwave reactor in an open-vessel mode. Hydrazide **7** was prepared from the acid **6** after its reaction with oxalyl chloride [13,18], followed by the treatment of the acyl chloride intermediate with hydrazine monohydrate. The hydrazide **7** was the common intermediate for the preparation of all azole derivatives by the reaction with different thioisocyanates, furnishing the carbothioamides **8a**–**8m** in good yields. These intermediates underwent basic promoted ring closure, generating the targeted triazoles (**9a**–**9m**) in moderate to good yields (Scheme 1). The literature describes that these reactions normally require conventional heating for several hours [34]. After some improvements in the experimental conditions, both reactions (preparation of carbothioamides and their ring closure to the corresponding triazoles) were conducted in a microwave reactor using water or ethanol as solvents, and resulting at reduced reaction times (30 min). Nowadays the principles of green chemistry are being observed in our laboratory, due to their growing importance, mostly for ecological and health reasons. Thus, chemical transformations have been conducted with the least expense of energy, decreased reaction times and, whenever possible, avoiding both the use of toxic solvents and harmful waste production [35].

The physical and spectroscopical data for the compounds **7**, **8a**–**8m** and **9a**–**9m** are described herein (Experimental section), while the spectral characterization of the isolated natural amide **1** and piperic acid (**6**) has been described previously [36,37]. The ^1^H-NMR analysis of the newly prepared triazoles **9a**–**9m** revealed characteristic absorptions assigned to the heterocyclic ring N-H protons at about δ 13 ppm that are quite different from absorptions assigned to the three different N-H protons present in the carbothioamides **8a**–**8m**, which showed signals between δ 7.65 to δ 10.24 ppm. The ^13^C-NMR spectra showed signals at about δ 168 ppm and δ 149 ppm, assigned to the two carbon atoms of heterocyclic ring (C_3_ and C_4_, respectively). The data observed in the high resolution mass spectra were consistent with the calculated values for the expected molecular formulas of the final products.

### 2.2. Biological Assays

All compounds were evaluated *in vitro* against the proliferative epimastigote form of Y strain *T. cruzi* having benznidazole (Bdz) as a control drug. The compounds **9c**, **9d**, **9e**, **9f** and **9m** which showed IC_50_ values below 30 μM on epimastigotes were further selected for cell viability assays on peritoneal macrophages (Mø) from Balb/c mice. The Mø were treated with the selected compounds described above at the concentrations of 0.5, 1.0, 2.5 and 5 μg mL^−1^. The number of viable cells was determined by a trypan blue dye exclusion assay after 72 h of treatment. In all the concentrations tested the five derivatives showed no significant toxicity on host cells, presenting cellular viability values ranging from 85% to 95%. The results obtained on the trypanocidal evaluation of the new triazoles are summarized in Table 1. 

**Table 1:** Toxic effects of new triazoles (**9a**–**9m**) on proliferative forms of *T. cruzi*.

Triazoles	IC_50_ (µM)	
	Epimastigotes	Amastigotes
9a	44.2 ± 7.30	*nt*
9b	50.16 ± 10.79	*nt*
9c	26.98 ± 6.98	11.11 ± 1.26
9d	13.22 ± 3.64	≥15.17 **
9e	15.38 ± 4.47	≥13.98 **
9f	18.30 ± 5.21	8.87 ± 2.39
9g	38.96 ± 8.88	*nt*
9h	39.39 ± 6.88	*nt*
9i	35.30 ± 6.26	*nt*
9j	50.75 ± 10.63	*nt*
9k	40.75 ± 10.50	*nt*
9l	48.02 ± 6.06	*nt*
9m	18.46 ± 3.95	9.59 ± 2.87
Benznidazole *	2.20 ± 0.16	2.50 ± 0.21

Notes:* Reference drug;** Maximum concentration allowed by trypan blue exclusion assay; *nt* = Not tested.

The *N*-alkyl-triazoles **9a** and **9b** had no important activity against the epimastigote form of *T. cruzi*, with IC_50_ values of 44.2 and 50.16 μM, respectively. With increasing length of the alkyl chain, the *N*-alkyl derivatives (**9c**–**9f**) exhibited a higher activity activity on epimastigotes, suggesting that lipophilicity is an important parameter for the trypanocidal activity of this family of triazoles interfering, for example, with their ability to cross cell membranes. In the *N*-benzyl and *N*-aryl series, the triazoles **9g**–**9l** showed no significant activity. Only **9m**, which has a *para*-trifluoromethylaryl substituent at *N*-4 of the heterocyclic ring, showed an important toxic effect against epimastigotes. In the evaluation against amastigotes of *T. cruzi* the compounds **9c**, **9f** and **9m** showed similar trypanocidal effects, with IC_50_ values of 11.11, 8.87 and 9.59 μM, respectively. The triazoles **9d** and **9e** showed higher IC_50_ values on amastigotes. Among the thirteen new compounds prepared, the triazole **9f** (*N*-cyclohexyl derivative, Scheme 1) had the best trypanocidal profile, presenting IC_50_ = 18.3 μM and 8.87 μM against epimastigotes and amastigotes of *Trypanosoma cruzi* (Y strain), respectively.

## 3. Experimental

### 3.1. General

Melting points were determined on a Büchi B-510 apparatus and are uncorrected. The ^1^H-NMR (500 MHz) and ^13^C-NMR (125 MHz) spectra were recorded on a Bruker Ultrashield Plus Spectrometer (BrukerBioSpin GmbH, Rheinstetten, Germany) operating at 500 MHz for ^1^H and 125 MHz for ^13^C. ^1^H and ^13^C-NMR shifts (δ) are reported in parts per million (ppm) with respect to DMSO-*d_6_* (2.50 ppm for ^1^H and 39.7 ppm for ^13^C). Chemical shifts (δ) were reported in ppm and coupling constants (*J*) in Hertz [Hz]. Signal multiplicity was assigned as singlet (s), doublet (d), doublet of doublets (dd), triplet (t), quartet (q), multiplet (m) and broad signal (bs). The low-resolution mass spectra were carried out on a Shimadzu GCMS-QP2010 Plus (Shimadzu Inc., Kyoto, Japan). Analytical conditions: Column: VF-5MS, 30 m × 0.25 mm × 0.25 μm (Varian Inc., Santa Clara, CA, USA); Column temperature: 200 °C for one minute and then increasing to 290 °C at a rate of 10 °C/min and holding for 40 minutes; Injector temperature: 270 °C. The high-resolution mass spectra were recorded with Q-TOF Micromass equipment (Waters, Milford, MA, USA). All the reactions involving microwave instrumentation used the Discover SP system (CEM Inc., Matthews, NC, USA) and were performed in open vessel mode. Analytical thin-layer chromatography (TLC) was performed on precoated silica gel plates (0.25 mm layer thickness) in an appropriate solvent and the spots were visualized under UV light (254 nm and 356 nm). The natural piperine (**1**) was extracted from powdered dry fruits of *P. nigrum* purchased from different sources on the market and presented physical and spectroscopic data consistent with that originally described [6,36,37]. The isolation method employed in this study was an adaptation of that previously reported [6].

#### 3.1.1. Microwave-assisted Hydrolysis of Natural Piperine: Preparation of 1-[(2*E*,4*E*)-5-(1,3-Benzodioxol-5-yl)penta-2,4-dienoyl]piperidine (**6**)

A suspension of piperine (**1**, 2.20 g, 7.72 mmol) in an 20% (w/v) ethanolic solution of NaOH (22 mL) was placeded in a 50 mL round bottom flask equipped with a magnetic stirring bar and a reflux apparatus. This suspension was then subjected to microwave irradiation (100 W) for 1 h in open vessel mode. The evolution of the reaction was monitored by TLC. At the end of the reaction the solvent was removed under reduced pressure and hot water was added to solubilize the carboxylate. The warm mixture was filtered and the resulting solution was acidified with concentrated HCl (37% w/v) until pH 3.0, whereupon the free acid precipitated as a yellow solid. After filtration the solid was washed twice with ice water. The acid **6** (1.37 g; 82%) was thus obtained as a yellow solid after recrystallization from ethanol and was characterized by its melting point, ^1^H- and ^13^C-NMR. All data were consistent with those previously reported in the literature [37].

#### 3.1.2. Synthesis of (2*E*,4*E*)-5-(1,3-Benzodioxol-5-yl)penta-2,4-dienohydrazide (**7**)

Acid **6** (500 mg, 2.29 mmol) and oxalyl chloride (1.5 mL, 17.60 mmol) were added to a 10 mL round bottom flask equipped with a magnetic stirring bar, rubber septum and kept under a dry N_2_ atmosphere. The resulting solution was subjected to stirring at room temperature for about 0.5 h. The evolution of the reaction was accompanied by TLC (indirectly by the reaction of an aliquot with methanol leading to the spontaneous formation of the corresponding methyl ester). After the removal of excess oxalyl chloride the resulting residue was dissolved in dry dichloromethane (5 mL). The resulting solution was added dropwise to an ice-cooled mixture of hydrazine monohydrate (1.0 mL, 20.64 mmol) and dichloromethane (7 mL) placed in a 25 mL round bottom flask equipped with stirring bar, rubber septum and kept under a dry N_2_ atmosphere. The reaction medium was allowed to reach room temperature (about thirty minutes) and then the solvent was removed under reduced pressure. The product precipitated with addition of ice water to the residue previously obtained and then was filtered, furnishing 375 mg (71% yield) of the hydrazide **7** as a yellow amorphous solid. m.p. = 237–239 °C. ^1^H-NMR δ (ppm): 9.30 (s, 1H, -NH-), 7.27 (d, 1H, *J* = 1.40 Hz), 7.19 (dd, 1H, *J* = 15.01 and 10.40 Hz), 7.00 (dd, 1H, *J* = 8.00 and 1.40 Hz), 6.86–6.97 (m, 3H), 6.05 (d, 1H, *J* = 15.10 Hz), 6.06 (s, 2H, -O-*CH_2_-*O-), 4.45 (s, 2H, -NH_2_); ^13^C-NMR δ (ppm): 165.23, 148.43, 148.19, 139.38, 138.26, 131.27, 125.82, 123.13, 123.05, 108.93, 106.10, 101.75.

#### 3.1.3. General Procedure for the Synthesis of Carbothioamides **8a** to **8m**

The suitable alkylisothiocyanates (0.517 mmol, 1.2 equiv.) were added to a suspension of hydrazide **7** (100 mg, 0.431 mmol) in ethanol (5 mL). The mixture was placed in a 10 mL round bottom flask equipped with a magnetic stirring bar and a reflux apparatus and then subjected to microwave irradiation (100 W) for 0.5 h in open vessel mode. The evolution of the reaction was monitored by TLC. After elimination of ethanol under reduced pressure and addition of water to the vessel the products were collected and recrystallized from ethanol. All compounds were characterized by ^1^H- and ^13^C-NMR. The yields, melting points and spectral data observed for each carbothioamide **8a**–**8m** prepared are as follows: 

*2-[(2E,4E)-5-(1,3-Bbenzodioxol-5-yl)penta-2,4-dienoyl]-N-methylhydrazinecarbothioamide* (**8a**). Brown amorphous solid, yield 67%, m.p. = 195–196 °C (ethanol). ^1^H-NMR δ (ppm): 9.89 (s, 1H, NH), 9.27 (s, 1H, NH), 7.98 (s, 1H, NH), 7.32 (s, 1H), 7.29 (dd, 1H, *J* = 14.98 and 10.56 Hz), 6.93–7.04 (m, 4H), 6.09 (d, 1H, *J* = 15.00 Hz), 6.07 (s, 2H, -O-*CH_2_*-O-), 2.87 (d, 3H, *J* = 15.00 Hz, -NH-*CH_3_*); ^13^C-NMR δ (ppm): 165.65, 148.46, 141.37, 139.53, 131.20, 125.53, 123.51, 122.40, 108.96, 106.18, 101.81, 31.40.

*2-[(2E,4E)-5-(1,3-Benzodioxol-5-yl)penta-2,4-dienoyl]-N-ethylhydrazinecarbothioamide* (**8b**). Brown amorphous solid, yield 90%, m.p. = 192–193 °C. ^1^H-NMR δ (ppm): 9.86 (s, 1H, NH), 9.18 (s, 1H, NH), 8.00 (t, 1H, *J* = 5.20 Hz, NH), 7.29 (d, 1H, *J* = 1.40 Hz), 7.25 (dd, 1H, *J* = 15.18 and 9.91 Hz), 7.00 (dd, 1H, *J* = 8.28 and 1.51 Hz), 6.91–6.99 (m, 3H), 6.08 (d, 1H, *J* = 15.30 Hz), 6.05 (s, 2H, -O-*CH_2_*-O-), 3.39-3.49 (m, 2H, -NH-*CH_2_*-CH_3_), 1.05 (t, 3H, *J* = 7.00 Hz, -NH-CH_2_-*CH_3_*); ^13^C-NMR δ (ppm): 181.65, 165.61, 148.42, 141.35, 139.46, 131.16, 125.48, 123.38, 122.36, 108.94, 106.20, 101.76, 38.96, 14.87.

*2-[(2E,4E)-5-(1,3-Benzodioxol-5-yl)penta-2,4-dienoyl]-N-isopropylhydrazinecarbothioamide* (**8c**). Pale yellow amorphous solid, yield 66%, m.p. = 193–194 °C. ^1^H-NMR δ (ppm): 9.82 (s, 1H, NH), 9.16 (s, 1H, NH), 7.67 (d, 1H, *J* = 7.80 Hz, NH), 7.31 (s, 1H), 7.26 (dd, 1H, *J* = 14.98 and 10.25 Hz), 6.93–7.03 (m, 4H), 6.10 (d, 1H, *J* = 15.00 Hz), 6.07 (s, 2H, -O-*CH_2_*-O-), 4.34–4.46 (m, 1H, -NH-*CH*-(CH_3_)_2_), 1.12 (d, 6H, *J* = 6.60 Hz, -NH-CH*-(CH_3_)_2_*); ^13^C-NMR δ (ppm): 165.49, 148.46, 141.20, 139.47, 131.22, 125.53, 123.48, 122.56, 108.96, 106.17, 101.81, 46.24, 22.39.

*2-[(2E,4E)-5-(1,3-Benzodioxol-5-yl)penta-2,4-dienoyl]-N-buthylhydrazinecarbothioamide* (**8d**). Yellow amorphous solid, yield 87%, m.p. = 173–175 °C. ^1^H-NMR δ (ppm): 9.87 (s, 1H, NH), 9.19 (s, 1H, NH), 8.00 (s, 1H, NH), 7.33 (s, 1H), 7.27 (dd, 1H, *J* = 14.82 and 10.72 Hz), 6.94–6.98 (m, 2H), 7.02–7.30 (m, 2H), 6.10 (d, 1H, *J* = 15.00 Hz), 6.08 (s, 2H,-O-*CH_2_*-O-), 3.48-3.44 (m, 2H, -NH-*CH_2_*-CH_2_-CH_2_-CH_3_), 1.49 (m, 2H, -NH-CH_2_-*CH_2_*-CH_2_-CH_3_), 1.28 (m, 2H, -NH-CH_2_-CH_2_-*CH_2_*-CH_3_), 0.90 (t, 3H, *J* = 6.90 Hz, NH-CH_2_-CH_2_-CH_2_-*CH_3_*); ^13^C-NMR δ (ppm): 165.57, 148.46, 148.40, 141.27, 139.49, 131.22, 125.53, 123.49, 122.49, 108.96, 106.17, 101.81, 43.82, 31.39, 19.92, 14.31.

*2-[(2E,4E)-5-(1,3-Benzodioxol-5-yl)penta-2,4-dienoyl]-N-hexylhydrazinecarbothioamide* (**8e**). Yellow amorphous solid, yield 90%, m.p. = 165–167 °C. ^1^H-NMR δ (ppm): 9.86 (s, 1H, NH), 9.18 (s, 1H, NH), 7.99 (s, 1H, NH), 7.32 (s, 1H), 7.26 (dd, 1H, *J* =14.98 and 10.56 Hz), 6.93–7.02 (m, 2H), 7.03–7.24 (m, 2H), 6.11 (d, 1H, *J* = 15.00 Hz), 6.07 (s, 2H, -O-*CH_2_*-O-), 3.41–3.42 (m, 2H, -NH-*CH_2_*-CH_2_-CH_2_-CH_2_-CH_2_-CH_3_), 1.48-1.66 (m, 2H, -NH-CH_2_-*CH_2_*-CH_2_-CH_2_-CH_2_-CH_3_), 1.27–1.37 (m, 6H, -NH-CH_2_-CH_2_-*CH_2_*-*CH_2_*-*CH_2_*-CH_3_), 0.88 (t, 3H, *J* = 5.00 Hz, -NH-CH_2_-CH_2_-CH_2_-CH_2_-CH_2_-*CH_3_*); ^13^C-NMR δ (ppm): 165.65, 164.17, 148.48, 141.35, 139.56, 131.30, 125.62, 123.57, 122.56, 109.04, 106.25, 101.88, 44.21, 31.62, 29.26, 26.48, 22.65, 14.50.

*2-[(2E,4E)-5-(1,3-Benzodioxol-5-yl)penta-2,4-dienoyl]-N-cyclohexylhydrazinecarbothioamide* (**8f**). Yellow amorphous solid, yield 91%, m.p. = 192–194 °C. ^1^H-NMR δ (ppm): 9.84 (s, 1H, NH), 9.18 (s, 1H, NH), 7.65 (d, 1H, NH, *J* = 2.52 Hz), 7.32 (s, 1H), 7.25 (dd, 1H, *J* = 14.98 and 10.56 Hz), 7.03 (d, 1H, *J* = 8.20 Hz), 6.93-7.04 (m, 3H), 6.10 (d, 1H, *J* = 15.13 Hz), 6.07 (s, 2H, -O-*CH_2_*-O-), 4.07–4.19 (bs, 1H, -NH-*CH*-(CH_2_-CH_2_)_2_-CH_2_), 1.06–1.89 (m,10H, -NH-CH-(*CH_2_-CH_2_*)*_2_-CH_2_*); ^13^C-NMR δ (ppm): 162.92, 148.39, 141.20, 139.46, 131.22, 125.54, 123.47, 122.55, 108.96, 106.17, 101.81, 53.37, 32.35, 25.67, 25.40.

*2-[(2E,4E)-5-(1,3-Benzodioxol-5-yl)penta-2,4-dienoyl]-N-phenylhydrazinecarbothioamide* (**8g**). Yellow amorphous solid, yield 87%, m.p. = 149–150 °C. ^1^H-NMR δ (ppm): 10.11 (s, 1H, NH), 9.74 (s, 1H, NH), 9.68 (s, 1H, NH), 7.44–7.48 (bs, 2H), 7.34–7.36 (m, 2H), 7.33 (s, 1H), 7.27–7.33 (m, 1H), 7.16–7.18 (m, 1H), 6.84-7.04 (m, 4H), 6.15 (d, 1H, *J* = 13.87 Hz), 6.07 (s, 2H, -O-*CH_2_*-O-); ^13^C-NMR δ (ppm): 148.46, 141.32, 139.70, 139.54, 131.22, 130.43, 128.47, 126.47, 125.56, 123.51, 122.53, 108.97, 106.18, 101.81.

*2-[(2E,4E)-5-(1,3-Benzodioxol-5-yl)penta-2,4-dienoyl]-N-benzylhydrazinecarbothioamide* (**8h**). Yellow amorphous solid, yield 84%, m.p. = 196–197 °C. ^1^H-NMR δ (ppm): 9.98 (s, 1H, NH), 9.41 (s, 1H, NH), 8.59 (bs, 1H, NH), 7.32 (s, 1H), 7.29–7.32 (m, 5H), 7.24–7.27 (m, 1H), 6.94–7.04 (m, 4H), 6.11 (d, 1H, *J* = 15.00 Hz), 6.07 (s, 2H, -O-*CH_2_*-O-), 4.74 (d, 2H, *J* = 5.30 Hz); ^13^C-NMR δ (ppm): 165.71, 148.41, 141.37, 139.94, 139.56, 131.21, 128.51, 127.60, 127.08, 125.53, 123.51, 122.45, 108.97, 106.18, 101.82, 41.17.

*2-[(2E,4E)-5-(1,3-Benzodioxol-5-yl)penta-2,4-dienoyl]-N-(3,4,5-trimethoxyphenyl) hydrazinecarbo-thioamide* (**8i**). Pale yellow amorphous solid, yield 77%, m.p. = 189–191 °C. ^1^H-NMR δ (ppm): 10.08 (s, 1H, NH), 9.67 (s, 1H, NH), 9.60 (s, 1H, NH), 7.32 (s, 1H), 7.27–7.32 (m, 1H), 7.03 (dd, 1H, *J* = 7.88 and 1.26 Hz), 6.86–7.06 (m, 3H), 6.91 (s, 2H), 6.16 (d, 1H, *J* = 11.03 Hz), 6.07 (s, 2H, -O-*CH_2_*-O-), 3.76 (s, 6H), 3.66 (s, 3H); ^13^C-NMR δ (ppm): 153.79, 148.46, 148.42, 141.42, 139.59, 135.40, 135.07, 131.21, 125.55, 123.51, 122.43, 108.97, 106.19, 104.42, 101.81, 60.56, 56.31.

*2-[(2E,4E)-5-(1,3-Benzodioxol-5-yl)penta-2,4-dienoyl]-N-tert-butylhydrazinecarbothioamide* (**8j**). Yellow amorphous solid, yield 80%, m.p. = 135–136 °C. ^1^H-NMR δ (ppm): 10.18 (s, 1H, NH), 9.95 (s, 1H, NH), 9.15 (s, 1H, NH), 7.30 (s, 1H), 7.24–7.30 (m, 1H), 7.02 (dd, 1H, *J* = 8.16 and 1.38 Hz), 6.92–6.99 (m, 3H), 6.13 (d, 1H, *J* = 14.81 Hz), 6.07 (s, 2H, -O-*CH_2_*-O-), 1.46 (s, 9H, -C-(*CH_3_*)_3_); ^13^C-NMR δ (ppm): 178.73, 169.55, 148.44, 141.54, 139.58, 131.17, 125.50, 123.48, 121.91, 108.94, 106.14, 101.81, 53.19, 29.09.

*2-[(2E,4E)-5-(1,3-Benzodioxol-5-yl)penta-2,4-dienoyl]-N-[4-(methylthio)phenyl] hydrazinecarbothio-amide* (**8k**). Yellow amorphous solid, yield 84%, m.p.= 175–177 °C. ^1^H-NMR δ (ppm): 10.24 (s, 1H, NH), 9.80 (s, 2H, NH), 7.55-7.64 (m, 1H), 7.55 (bs, 1H), 7.36-7.45 (m, 4H),7.06–7.18 (m, 4H), 6.27 (d, 1H, *J* = 14.10 Hz), 6.20 (s, 2H, -O-*CH_2_*-O-), 2.65 (s, 3H, -S-*CH_3_*); ^13^C-NMR δ (ppm): 148.48, 141.36, 139.57, 136.96, 131.24, 126.30, 125.57, 123.52, 122.53, 121.67, 108.99, 106.22, 101.83, 15.63.

*2-[(2E,4E)-5-(1,3*-*Benzodioxol-5-yl)penta-2,4-dienoyl]-N-(3-methoxyphenyl) hydrazinecarbothio-amide* (**8l**). Dark green amorphous solid, yield 65%, m.p.= 174–175 °C. ^1^H-NMR δ (ppm): 10.09 (s, 1H, NH), 9.70 (s, 2H, NH), 7.32 (s, 1H), 7.22–7.38 (m, 3H), 6.93–7.08 (m, 5H), 6.74 (d, 1H, *J* = 8.03 Hz), 6.13 (d, 1H, *J* = 15.30 Hz), 6.07 (s, 2H, -O-*CH_2_*-O-), 3.75 (s, 3H); ^13^C-NMR δ (ppm): 181.15, 172.77, 164.55, 148.45, 141.33, 140.79, 139.54, 131.19, 131.13, 125.53, 123.50, 122.45, 118.68, 114.96, 111.62, 108.94, 106.15, 101.79, 55.54.

*2-[(2E,4E)-5-(1,3-Benzodioxol-5-yl)penta-2,4-dienoyl]-N-[4-(trifluoromethyl)phenyl] hydrazinecarbo-thioamide* (**8m**). Pale yellow amorphous solid, yield 83%, m.p. = 177–178 °C. ^1^H-NMR δ (ppm): 10.18 (s, 1H, NH), 10.15 (s, 1H, NH), 9.93 (s, 1H, NH), 7.69–7.80 (m, 4H), 7.33 (s, 1H), 7.27–7.34 (m, 1H), 6.93–7.08 (m, 4H), 6.16 (d, 1H, *J* = 14.80 Hz), 6.07 (s, 2H, -O-*CH_2_*-O-); ^13^C-NMR δ (ppm): 181.27, 164.55, 148.45, 143.54, 141.54, 139.69, 131.16, 130.10, 125.49, 124.81 (q, CF_3_, *J*_C-F_ = 270 Hz), 123.53, 122.28, 121.60, 117.13, 108.94, 106.16, 101.80.

#### 3.1.4. General Procedure to the Synthesis of Triazoles **9a** to **9m**

In a 10 mL bottom rounded flask equipped with a magnetic stirring bar and a reflux apparatus were placed the corresponding hydrazine carbothioamide **8a**–**8m** (1.37 mmol) and an aqueous solution of NaOH (2 mL, 1 equiv.). This mixture was then subjected to microwave irradiation (100 W) for 0.5 h in open vessel mode. The evolution of the reaction was monitored by TLC. Then the mixture was acidified with a 10% aqueous solution of HCl (w/v) until pH 3, filtered and the solids were washed twice with ice water (5 mL). After recrystallization from ethanol all the triazoles were characterized by NMR and low- and high-resolution mass spectra. All compounds were characterized by ^1^H- and ^13^C- NMR. The yields, melting points and spectral data observed for each carbothioamide **9a**–**9m** prepared are as follows:

*5-[(1E,3E)-4-(1,3-Benzodioxol-5-yl)buta-1,3-dien-1-yl]-4-methyl-2,4-dihydro-3H-1,2,-triazole-3-thione* (**9a**). Brown amorphous solid, yield 71%, m.p. = 220–222 °C. ^1^H-NMR δ (ppm): 13.78 (s, 1H, NH), 7.28 (dd, 1H, *J* = 15.40 and 11.00 Hz), 7.23 (s, 1H), 6.90–7.07 (m, 4H), 6.54 (d, 1H, *J* = 15.40 Hz), 6.07 (s, 2H, -O-*CH_2_-*O-), 3.39 (s, 3H, -N-*CH_3_*); ^13^C-NMR δ (ppm): 167.11, 150.15, 148.45, 148.24, 137.55, 137.02, 131.31, 126.50, 123.00, 113.95, 109.03, 105.91, 101.77, 30.40; MS (*m/z*): 287; HRMS (ES)-m/z: [M+H]^+^, found 288.0807. C_15_H_15_N_3_O_2_S requires 288.0806.

*5-[(1E,3E)-4-(1,3-Benzodioxol-5-yl)buta-1,3-dien-1-yl]-4-ethyl-2,4-dihydro-3H-1,2,4-triazole-3-thione* (**9b**). Brown amorphous solid, yield 62%, m.p. = 208–209 °C. ^1^H-NMR δ (ppm): 13.76 (s, 1H, NH), 7.28 (dd, 1H, *J* = 15.30 and 10.70 Hz), 7.20 (s, 1H), 6.88–7.07 (m, 4H), 6.57 (d, 1H, *J* = 15.30 Hz), 6.05 (s, 2H, -O-*CH_2_*-O-), 4.08 (q, 2H, *J* = 7.03 Hz, -N-*CH_2_*-CH_3_), 1.19 (t, 3H, *J* = 7.03 Hz, -NH-CH_2_-*CH_3_*); ^13^C-NMR δ (ppm): 166.46, 149.55, 148.44, 148.23, 137.56, 137.21, 131.32, 126.50, 122.95, 113.50, 109.02, 105.90, 101.75, 38.33, 14.22; MS (*m/z*): 301; HRMS (ES)-m/z: [M+H]^+^, found 302.0963. C_15_H_15_N_3_O_2_S requires 302.0963.

*5-[(1E,3E)-4-(1,3-Benzodioxol-5-yl)buta-1,3-dien-1-yl]-4-isopropyl-2,4-dihydro-3H-1,2,4-triazole-3-thione* (**9c**). Pale yellow amorphous solid, yield 90%, m.p. = 210–211 °C. ^1^H-NMR δ (ppm): 13.78 (s, 1H, NH), 7.15-7.27 (m, 2H), 7.20 (s, 1H), 6.87–7.00 (m, 3H), 6.75 (d, 1H, *J* = 15.00 Hz), 6.07 (s, 2H, -O-*CH_2_*-O-), 5.09-5.14 (m, 1H, -NH-*CH*-(CH_3_)_2_), 1.47 (d, 6H, *J* = 5.00 Hz, -NH-CH-(*CH_3_*)_2_); ^13^C-NMR δ (ppm): 166.46, 149.57, 148.46, 148.22, 137.23, 131.44, 126.77, 122.96, 114.55, 109.06, 105.84, 101.81, 46.61, 20.78; MS (*m/z*): 315; HRMS (ES)-m/z: [M+H]^+^, found 316.1120. C_16_H_17_N_3_O_2_S requires 316.1119.

*5-[(1E,3E)-4-(1,3-Benzodioxol-5-yl)buta-1,3-dien-1-yl]-4-butyl-2,4-dihydro-3H-1,2,4-triazole-3-thione* (**9d**). Yellow amorphous solid, yield 71%, m.p. = 173–174 °C. ^1^H-NMR δ (ppm): 13.78 (s, 1H, NH), 7.29 (dd, 1H, *J* = 15.10 and 11.00 Hz), 7.22 (s, 1H), 6.89–7.09 (m, 4H), 6.58 (d, 1H, *J* = 15.10 Hz), 6.07 (s, 2H, -O-*CH_2_*-O-), 4.04 (t, 2H, *J* = 6.78 Hz, -NH-*CH_2_*-CH_2_-CH_2_-CH_3_), 1.60–1.63 (m, 2H, -NH-CH_2_-*CH_2_*-CH_2_-CH_3_), 1.31-1.36 (m, -NH-CH_2_-CH_2_-*CH_2_*-CH_3_), 0.93 (t, 3H, *J* = 7.20 Hz, -NH-CH_2_-CH_2_-CH_2_-*CH_3_*); ^13^C-NMR δ (ppm): 166.74, 149.79, 148.48, 148.27, 137.58, 137.17, 131.55, 125.53, 126.55, 123.06, 113.56, 109.06, 105.89, 101.81, 42.92, 30.87, 19.78, 14.12; MS (*m/z*): 329; HRMS (ES)-m/z: [M+H]^+^, found 330.1276. C_17_H_19_N_3_O_2_S requires 330.1276.

*5-[(1E,3E)-4-(1,3-Benzodioxol-5-yl)buta-1,3-dien-1-yl]-4-hexyl-2,4-dihydro-3H-1,2,4-triazole-3-thione* (**9e**). Dark green amorphous solid, yield 79%, m.p. = 124–125 °C. ^1^H-NMR δ (ppm): 13.77 (s, 1H, NH), 7.27–7.32 (m, 1H), 7.22 (s, 1H), 6.89–7.06 (m, 4H), 6.57 (d, 1H, *J* = 15.45 Hz), 6.07 (s, 2H, -O-*CH_2_*-O-), 4.03 (t, 2H, *J* = 7.57 Hz, -NH-*CH_2_*-CH_2_-CH_2_-CH_2_-CH_2_-CH_3_), 1.62–1.64 (m, 2H, -NH-CH_2_-*CH_2_*-CH_2_-CH_2_-CH_2_-CH_3_), 1.27–1.30 (m, 6H, -NH-CH_2_-CH_2_-*CH_2_*-*CH_2_*-*CH_2_*-CH_3_), 0.84–0.90 (m, 3H, -NH-CH_2_-CH_2_-CH_2_-CH_2_-CH_2_-*CH_3_*); ^13^C-NMR δ (ppm): 166.73, 149.79, 148.48, 137.58, 137.19, 131.35, 126.54, 123.06, 113.56, 109.07, 105.89, 101.81, 43.09, 31.26, 28.66, 26.06, 22.47, 14.36; MS (*m/z*): 357; HRMS (ES)-m/z: [M+H]^+^, found 358.1589. C_19_H_23_N_3_O_2_S requires 358.1589.

*5-[(1E,3E)-4-(1,3-Benzodioxol-5-yl)buta-1,3-dien-1-yl]-4-cyclohexyl-2,4-dihydro-3H-1,2,4-triazole-3-thione* (**9f**). Yellow amorphous solid, yield 91%, m.p. = 192–194 °C. ^1^H-NMR δ (ppm): 13.76 (s, 1H, NH), 7.19 (d, 1H, *J* = 1.89 Hz), 7.19–7.24 (m, 1H), 7.11–7.17 (m, 1H), 6.99 (dd, 1H, *J* = 10.00 and 5.00 Hz), 6.93 (d, 1H, *J* = 8.20 Hz), 6.85 (d, 1H, *J* = 15.13 Hz), 6.75 (d, 1H, *J* = 14.50 Hz), 6.05 (s, 2H, -O-*CH_2_*-O-), 4.55–4.75 (bs, 1H, -NH-*CH*-(CH_2_-CH_2_)_2_-CH_2_), 1.33–2.08 (m, 10H, -NH-CH-(*CH_2_-CH_2_*)*_2_-CH_2_*); ^13^C-NMR δ (ppm): 166.63, 149.63, 148.47, 148.21, 141.20, 137.22, 131.45, 126.80, 122.97, 114.63, 109.06, 105.82, 101.81, 55.45, 32.35, 26.13, 24.87; MS (*m/z*): 355; HRMS (ES)-m/z: [M+H]^+^, found 356.1433. C_19_H_21_N_3_O_2_S requires 356.1432.

*5-[(1E,3E)-4-(1,3-Benzodioxol-5-yl)buta-1,3-dien-1-yl]-4-phenyl-2,4-dihydro-3H-1,2,4-triazole-3-thione* (**9g**). Yellow amorphous solid, yield 89%, m.p. = 244–245 °C. ^1^H-NMR δ (ppm): 14.00 (s, 1H, NH), 7.58–7.65 (m, 3H), 7.44 (dd, 2H, *J* = 8.04 and 1.42 Hz), 7.20 (d, 1H, *J* = 1.58 Hz), 7.15 (dd, 1H, *J* = 15.00 and 10.00 Hz), 6.94–7.00 (m, 2H), 6.89 (d, 1H, *J* = 7.88 Hz), 6.78 (d, 1H, *J* = 15.45 Hz), 6.04 (s, 2H, -O-*CH_2_*-O-) 5.94 (d, 1H, *J* = 15.45 Hz);^13^C-NMR δ (ppm): 168.03, 149.83, 148.25, 137.79, 137.37, 134.02, 131.31, 130.10, 129.06, 126.54, 123.29, 113.40, 108.93, 105.91, 101.75; MS (*m/z*): 349; HRMS (ES)-m/z: [M+H]^+^, found 350.0963. C_19_H_15_N_3_O_2_S requires 350.0963.

*5-[(1E,3E)-4-(1,3-Benzodioxol-5-yl)buta-1,3-dien-1-yl]-4-benzyl-2,4-dihydro-3H-1,2,4-triazole-3-thione* (**9h**). Yellow amorphous solid, yield 76%, m.p. = 187–188 °C. ^1^H-NMR δ (ppm): 13.97 (s, 1H, NH), 7.37–7.40 (m, 2H), 7.28–7.32 (m, 3H), 7.21 (d, 1H, *J* = 1.26 Hz), 7.21–7.26 (m, 1H), 6.93 (d, 1H, *J* = 15.45 Hz), 6.93–6.99 (m, 2H), 6.85 (d, 1H, *J* = 15.45 Hz), 6.42 (d, 1H, *J* = 15.45 Hz), 6.06 (s, 2H, -O-*CH_2_*-O-), 5.35 (s, 2H); ^13^C-NMRδ (ppm): 167.49, 150.06, 148.30, 137.94, 137.38, 136.42, 131.24, 129.24, 128.21, 127.37, 126.35, 123.20, 113.40, 109.01, 105.95, 101.80, 45.92; MS (*m/z*): 363; HRMS (ES)-m/z: [M+H]^+^, found 364.1120. C_20_H_17_N_3_O_2_S requires 364.1119.

*5-[(1E,3E)-4-(1,3-Benzodioxol-5-yl)buta-1,3-dien-1-yl]-4-(3,4,5-trimethoxyphenyl)-2,4-dihydro-3H-1,2,4-triazole-3-thione* (**9i**). Pale yellow amorphous solid, yield 81%, m.p. = 242–243 °C. ^1^H-NMR δ (ppm): 13.94 (s, 1H, NH), 7.21 (d, 1H, *J* = 1.58 Hz), 7.17–7.22 (m, 1H), 7.03 (dd, 1H, *J* = 15.00 and 10.00 Hz), 6.96 (dd, 1H, *J* = 10.00 and 5.00 Hz), 6.90 (d, 1H, *J* = 7.88 Hz), 6.81 (d, 1H, *J* = 15.00 Hz), 6.80 (s, 2H), 6.03 (d, 1H, *J* = 15.00 Hz), 6.04 (s, 2H, -O-*CH_2_*-O-), 3.80 (s, 6H), 3.78 (s, 3H); ^13^C-NMR δ (ppm): 168.01, 153.73, 150.06, 148.43, 148.22, 138.41, 137.58, 137.16, 131.39, 129.53, 126.74, 123.23, 113.65, 108.94, 106.85, 105.89, 101.75, 60.59, 56.77; MS (*m/z*): 439; HRMS (ES)-m/z: [M+H]^+^, found 440.1280. C_22_H_21_N_3_O_5_S requires 440.1280.

*5-[(1E,3E)-4-(1,3-Benzodioxol-5-yl)buta-1,3-dien-1-yl]-4-tert-butyl-2,4-dihydro-3H-1,2,4-triazole-3-thione* (**9j**). Yellow amorphous solid, yield 50%, m.p. = 183–184 °C. ^1^H-NMR δ (ppm): 7.63 (s, 1H, NH), 7.22 (s, 1H), 6.84 (d, 1H, *J* = 15.06 Hz), 6.89–7.04 (m, 4H), 6.48 (d, 1H, *J* = 15.31 Hz), 6.06 (s, 2H, -O-*CH_2_*-O-), 1.36 (s, 9H, -C-(*CH_3_*)_3_); ^13^C-NMR δ (ppm): 161.97, 157.91, 148.42, 148.12, 136.76, 135.29, 131.41, 126.51, 122.87, 113.17, 109.00, 105.87, 101.76, 51.50, 28.79; MS (*m/z*): 329; HRMS (ES)-m/z: [M+H]^+^, found 330.1276. C_17_H_20_N_3_O_2_S requires 330.1276.

*5-[(1E,3E)-4-(1,3-Benzodioxol-5-yl)buta-1,3-dien-1-yl]-4-[4-(methylthio)phenyl]-2,4-dihydro-3H-1,2,4-triazole-3-thione* (**9k**). Pale yellow amorphous solid, yield 75%, m.p. = 209–211 °C. ^1^H-NMR δ (ppm): 13.98 (s, 1H, NH), 7.47 (d, 2H, *J* = 10.00 Hz), 7.37 (d, 2H, *J* = 10.00 Hz), 7.20 (d, 1H, *J* = 1.00 Hz), 7.16–7.22 (m, 1H), 7.00 (dd, 1H, *J* = 15.00 and 10.00 Hz), 6.95 (dd, 1H, *J* = 10.00 and 5.00 Hz), 6.90 (d, 1H, *J* = 8.03 Hz), 6.81 (d, 1H, *J* = 15.31 Hz), 6.04 (s, 2H, -O-*CH_2_*-O-), 5.95 (d, 1H, *J* = 15.56 Hz), 2.52 (s, 3H, -S-*CH_3_*); ^13^C-NMR δ (ppm): 148.48, 141.36, 139.57, 136.96, 131.24, 126.30, 125.57, 123.52, 122.53, 121.67, 108.99, 106.22, 101.83, 15.63; MS (*m/z*): 396; HRMS (ES)-m/z: [M+H]^+^, found 396.0840. C_20_H_18_N_3_O_2_S_2_ requires 396.0840.

*5-[(1E,3E)-4-(1,3-Benzodioxol-5-yl)buta-1,3-dien-1-yl]-4-(3-methoxyphenyl)-2,4-dihydro-3H-1,2,4-triazole-3-thione* (**9l**). Yellow amorphous solid, yield 80%, m.p. = 248–249 °C. ^1^H-NMR δ (ppm): 13.97 (s, 1H, NH), 7.53 (t, 1H, *J* = 8.16 Hz), 7.20 (d, 1H, *J* = 1.51 Hz), 7.13–7.18 (m, 2H), 7.04–7.05 (m, 1H), 6.95–6.99 (m, 3H), 6.90 (d, 1H, *J* = 8.03 Hz), 6.80 (d, 1H, *J* = 15.56 Hz), 5.97 (d, 1H, *J* = 15.31 Hz), 6.04 (s, 2H, -O-*CH_2_*-O), 3.83 (s, 3H); ^13^C-NMR δ (ppm): 167.62, 160.36, 149.81, 148.40, 148.22, 137.71, 137.27, 135.00, 131.32, 130.87, 126.57, 123.23, 121.07, 115.74, 114.84, 113.42, 108.91, 105.88, 101.73, 56.01; MS(*m/z*): 379; HRMS (ES)-m/z: [M+H]^+^, found 380.1069. C_20_H_18_N_3_O_3_S requires 380.1068.

*5-[(1E,3E)-4-(1,3-Benzodioxol-5-yl)buta-1,3-dien-1-yl]-4-[4-(trifluoromethyl)phenyl]-2,4-dihydro-3H-1,2,4-triazole-3-thione* (**9m**). Brown amorphous solid, yield 70%, m.p. = 232–233 °C. ^1^H-NMR δ (ppm): 14.09 (s, 1H, NH), 8.03 (d, 2H, *J* = 8.53Hz), 7.75 (d, 2H, *J* = 8.28 Hz), 7.20 (d, 1H, *J* = 1.51 Hz), 7.13–7.18 (m, 1H), 6.95–7.00 (m, 2H), 6.90 (d, 1H, *J* = 8.03Hz), 6.83 (m, 1H), 5.96 (d, 1H, *J* = 15.31 Hz), 6.04 (s, 2H, -O-*CH_2_*-O-); ^13^C-NMR δ (ppm): 167.78, 149.61, 148.41, 148.27,137.87, 137.70, 137.55, 131.29, 130.29, 130.30, 127.04 (q, *J*_C-F_ = 270.00 Hz)127.19, 126.51, 123.26, 113.14, 108.93, 105.82, 101.75; MS(*m/z*): 417; HRMS (ES)-m/z: [M+H]^+^, found 418.0837. C_20_H_15_F_3_N_3_O_2_S requires 418.0837.

### 3.2. Biological Assays

#### 3.2.1. Parasites

*Trypanosoma cruzi* (Y strain) was obtained from the Fundação Oswaldo Cruz (Rio de Janeiro, Brazil) culture collection, and maintained in the Laboratory of Glycobiology (UFRJ, Rio de Janeiro, Brazil) facilities. Epimastigote forms were maintained by weekly transfers at 28 °C in brain-heart-infusion (BHI) medium (Acumedia, Lansing, MI, USA), supplemented with 10 mg of hemin (Sigma, St. Louis, MO, USA), 20 mg of folic acid L^−1^ (Sigma) and 10% heat-inactivated fetal calf serum (FCS) (BHI-FCS medium, Gibco, Grand Island, NY, USA). Tissue culture-derived trypomastigotes were obtained after infection of confluent monolayers of Vero cells (kidney epithelial cells extracted from African green monkeys) with blood trypomastigotes (Y strain) to establish the intracellular cycle and maintained in RPMI 1640 medium containing 10% FCS under an atmosphere of 5% CO_2_ at 37 °C. The tissue culture-derived trypomastigotes were used to infect Mø *in vitro* to evaluate the toxic effects of the drugs [14].

#### 3.2.2. Anti-Epimastigote Effect

For evaluation of the anti-epimastigote effects, parasites (1 × 10^5^ cells per mL) were subcultured in the absence or presence of different concentrations (0.5 to 25 μg.mL^−1^) of the drugs **9a**–**9m**, from a 5 mg mL^−1^ stock solution in DMSO (Sigma). The parasites were incubated in BHI-FCS medium with or without drugs in a final volume of 1.0 mL in 24-well plates (TPP, Trasadingen, Switzerland), and after 7 days, the toxic effects of the drugs were quantified by the direct count of the live epimastigotes in a Neubauer chamber [14]. Drug-free medium contained comparable final concentration of DMSO (0.05%) has been used as control.

#### 3.2.3. Cytotoxicity to Macrophages

The evaluation of the toxic effects of the compounds was carried out as previously described [18]. Murine peritoneal Mø were seeded (1 × 10^6^ cells per well) in 24-well plates with 1 mL of RPMI medium containing 10% FCS. The cells were allowed to attach for 24 h at 37 °C and then exposed for 72 h to the five compounds (**9c**, **9d**, **9e**, **9f** and **9m**) which showed highest activity against epimastigote forms (maximum final concentration of DMSO was 0.05%). The five drugs were assayed in increasing concentrations (0.5, 1.0, 2.5, 5.0 µg mL^−1^). Afterwards, the cells were washed with PBS, and RPMI was added to the culture before the addition of vital dye trypan blue in a final concentration of 0.01%. The toxic effects of the drugs were monitored by the count of 200 cells in a Neubauer chamber where plasma membrane permeability was evaluated [13,18].

#### 3.2.4. Anti-Amastigote Effect

Resident peritoneal Mø from non-infected BALB/c mice were harvested and cultured as described above. Adhered Mø were infected with *T. cruzi* trypomastigotes at a 10:1 parasite/macrophage ratio and incubated at 37 °C under 5% CO_2_ for 1 h. Subsequently, non-infective cells were removed by extensive washing with PBS, and the infected macrophage cultures were treated with increasing concentrations (0.5, 1.0, 2.5, 5.0 µg mL^−1^) of the compounds **9c**, **9d**, **9e**, **9f** and **9m** which showed anti-epimastigote activity and non-toxic effects on Mø at the same concentrations assayed. After, monolayers were washed with PBS at 37 °C, fixed in methanol, and stained with Giemsa (Sigma). The amastigote survival was determined by the counting of 200 cells in triplicate, the percentage of infected cells was analyzed, as well as the number of amastigotes per macrophage and the endocytic index of the infected cells [13,18]. 

#### 3.2.5. Statistical Analysis

The 50% inhibitory concentrations (IC_50_) values shown in the Table 1 represent the mean of experiments carried out in triplicate. The IC_50_ of all compounds were determined by linear regression analysis using the program IGOR Pro 2.03 (WaveMetrics, Inc, Lake Oswego, OR, USA).

#### 3.2.6. Ethics Statement

All animal procedures were approved by the animal-care ethics committee of the Centro de Ciências da Saúde/UFRJ (License #DAHEICB 055) and were performed under the guidelines from SBCAL (Brazilian Society of Science in Laboratory Animals) and strictly followed the Brazilian law for Procedures for the Scientific Use of Animals (11.794/2008).

## 4. Conclusions

The results shown herein highlight the potential use of natural piperine as a precursor for new molecules suitable for the treatment of Chagas’s disease. Through the use of bioisosterism and molecular hybridization as molecular modification strategies we were able to design and synthesize a new series of 1,2,4-triazole-2-thiones, derivatives of the natural amide piperine, which showed activity against proliferative forms of *T. cruzi*. The cyclohexyl triazole derivative **9f** showed the best activity profile, presenting IC_50_ = 18.3 and 8.87 μM, against epimastigotes and amastigotes of *Trypanosoma cruzi*, respectively, and low toxic effects on host cells (murine macrophages).
